# A Novel Strain of *Orientia tsutsugamushi* Detected from Chiggers (Acari: Trombiculidae) on Wild Rodents

**DOI:** 10.3390/pathogens14010029

**Published:** 2025-01-03

**Authors:** Hak Seon Lee, Seong Yoon Kim, Hee Il Lee

**Affiliations:** Division of Vectors and Parasitic Diseases, Korea Disease Control and Prevention Agency, 187 Osongsaengmyeong 2-ro, Osong-eup, Heungdeok-gu, Cheongju 28159, Chungbuk, Republic of Korea; hslee8510@korea.kr (H.S.L.); gunbo0402@korea.kr (S.Y.K.)

**Keywords:** *Orientia tsutsugamushi*, novel strain, chigger mites

## Abstract

Scrub typhus is caused by intracellular bacteria belonging to the genus *Orientia*. Until 2010, the endemic region was thought to be restricted to the Asia–Pacific region. *Orientia* species have recently been discovered in South America, Africa, Europe, and North America. In accordance with these circumstances, we tried to find new or novel bacterial strains in the Republic of Korea (ROK). We found that a new strain of *O. tsutsugamushi* formed a unique clade based on a 56-kDa type-specific antigen gene and showed 63.2–77.8% similarity to other strains of the same species. Additionally, we identified another sequence with 99.8% similarity to the O3 strain, which has not been recorded in the ROK and whose pathogenicity remains unknown. These findings confirm the diversity of *O. tsutsugamushi* strains in the ROK, and highlight the need for continued surveillance and further studies to characterize the pathogenicity of this novel bacterial strain.

## 1. Introduction

The genus *Orientia* is an intracellular gram-negative bacterium that causes scrub typhus in humans [1]. Chigger mites, a larval stage of the family Trombiculidae, are known vectors and reservoirs of *Orientia* [2,3]. *Orientia* is maintained in nature through transstadial and transovarial transmission in chigger mites [1,3]. Until 2010, only one species in the genus *Orientia* was known, and its distribution was limited to an endemic area called the Tsutsugamushi Triangle, which includes the Republic of Korea (ROK), Japan, China, Thailand, Indonesia, Malaysia, Pakistan, northern Australia, and the islands of the western Pacific and Indian Oceans [3,4,5,6,7]. However, Izzard et al. [8] recorded a new species, *Candidatus* Orientia chuto, in a patient with scrub typhus in Dubai with molecular characterization. Balcells et al. [9] reported a case of scrub typhus that occurred on the island of Chiloe in southern Chile and confirmed through molecular analysis that it was not identical to the *Orientia tsutsugamushi* distributed in Asia. In 2015, Cosson et al. [10] reported that 16S rRNA sequences similar to those of *Orientia* were identified from wild rodents collected from Africa and Europe. Masakhwe et al. [11] reported *Candidatus* O. chuto in chigger mites collected from wild rodents in Kenya, Africa. Abarca et al. [12] recorded a novel species causing scrub typhus in Chile, with a molecular description designated as *Candidatus* O. chiloeensis. Recently, the 16S rRNA gene sequence of *Orientia* was detected in free-living chiggers collected in North Carolina, USA [13]. Similarly, *Orientia* was discovered outside its traditional endemic distribution, expanding its distribution range.

Up to now, only *O. tsutsugamushi* has been found in the ROK. Therefore, we did not consider the possibility of the distribution of a new species or make efforts to detect new species or other strains. However, it is necessary to re-examine the distribution of *Orientia* in the ROK according to international circumstances. This study aimed to determine whether new or novel bacterial strains exist in the ROK as a result of the global expansion of the distribution of *Orientia* caused by trombiculid mites.

## 2. Materials and Methods

### 2.1. Ethics Statement

This study was approved by the Institutional Animal Care and Use Committee (IACUC) of Korea Disease Control and Prevention Agency (No. KDCA-003-21) and followed the guidelines for ethical procedures on the use of animals.

### 2.2. Trapping and Sampling

**Figure 1:**
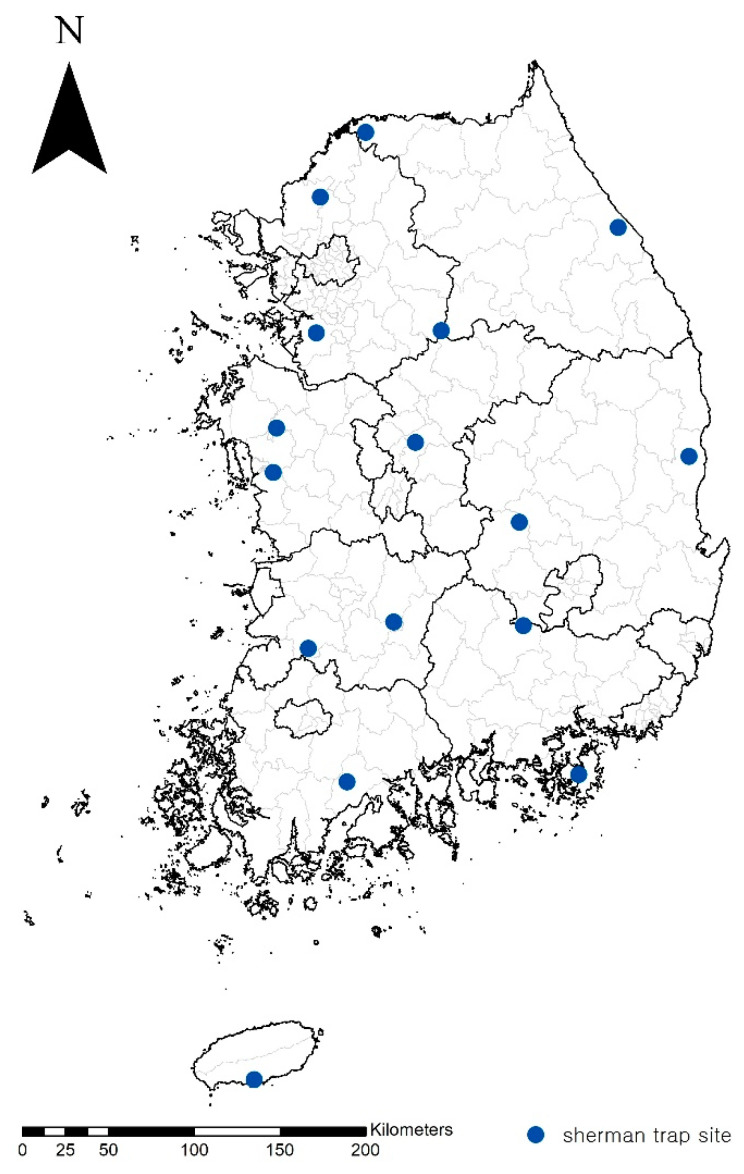
Collection sites of wild rodents.

Wild rodents were collected from 16 sites nationwide in the fourth week of October 2022, as described in our previous study (Figure 1) [14]. The captured rodents were euthanized by carbon dioxide (CO_2_) and hung for 24 h on a Petri dish filled with tap water. Subsequently, the fallen trombiculid mites were harvested from the surface of the water. The trombiculid mites were stored in 70% ethanol at −20 °C for further study. Approximately 10% of the chiggers were randomly selected for further identification and molecular analysis.

### 2.3. Chigger Identification, Pooling, and Total DNA Extraction

Approximately 10% of the trombiculid mites from wild rodents collected in 2022 were identified under an Olympus BX-43 fluorescence optical microscope with a U-FBWA fluorescence mirror unit (for fluorescent-isothiocyanate staining), Hyper E630 light emitting diode illuminator, DP23 microscope digital camera, and cellSens version 4.2 (EVIDENT Corp., Tokyo, Japan). These settings were adopted from the autofluorescence microscopy method proposed by Kumlert et al. [15]. Trombiculid mites were identified at the species level based on their morphological characteristics using the taxonomic keys proposed by Ree [16]. The identified specimens were pooled into 10 individuals of the same species in prefilled bead lysis tubes (INVIRUSTECH Co., Inc., Gwangju, Republic of Korea). Then, the pools were homogenized using a Precellys Evolution (Bertin Technologies, Bretonneux, France) with 400 µL of phosphate-buffered saline. Genomic DNA was extracted from all homogenized solutions using the KingFisher Flex system (Thermo Fisher Scientific, Waltham, MA, USA) with the MagMAX DNA Multi-Sample Ultra 2.0 Kit (Applied Biosystems, Waltham, MA, USA), according to the manufacturer’s protocol.

### 2.4. PCR Assays for Screening and Detecting Orientia DNA

To screen the DNA of *Orientia*, a genus-specific quantitative real-time PCR (qPCR) assay designed by Jiang et al. [17] was used to target the 16S rRNA (*rrs*) gene. We optimized the method using PowerAmp Real-time PCR Master Mix II (KogeneBiotech Co., Ltd., Seoul, Republic of Korea) and the Quantstudio 5 (Thermo Fisher Scientific, Inc., Waltham, MA, USA) thermocycler instrument and qPCR with primer at 0.5 µM, probe at 0.15 µM, initial denaturation (including enzyme activation) at 95 °C for 10 min, 45 cycles of denaturation at 95 °C for 15 s, and annealing/elongation at 58 °C for 1 min. The total volume of each reaction mixture was 20 µL, and contained 1 µL of template DNA.

Positive DNA samples in the screening assay were used to amplify the 56-kDa type-specific antigen (56-kDa *tsa*) and 47-kDa high-temperature requirement A (47-kDa *htrA*) genes for the molecular characterization of genes in *Orientia* species. In addition, *O. tsutsugamushi*-positive DNA samples confirmed during national surveillance of trombiculid mites from 2022 to 2023 were also used (Table 1). These samples were recognized as Kato-related strains of *O. tsutsugamushi* but could not be clearly distinguished.

To amplify these genes, nested conventional PCR for the 56-kDa *tsa* and semi-nested conventional PCR for the 47-kDa *htrA* were conducted using previously described primers and conditions (Table 2) [11,12]. All PCR reactions were performed with the AccuPower PCR PreMix (Bioneer Corp., Daejeon, Republic of Korea) in 20 µL volumes. The first and second PCR mixture contained 2 µL of template DNA and 1 µL of the first PCR product, respectively. The amount of ultrapure water (BIOSESANG, Yongin, Republic of Korea) was adjusted to match the total volume of the PCR mixture. The ProFlex PCR System (Applied Biosystems, Waltham, MA, USA) thermal cycler was used for amplification, and the second PCR amplicons were confirmed using the QIAxcel Advanced System with the QIAxcel DNA screening kit (2400) (QIAGEN GmbH, Hilden, Germany).

### 2.5. Nucleotide Sequencing and Phylogenetic Analysis

We used a commercial service (Cosmogenetech Co., Ltd., Seoul, Republic of Korea) to purify and sequence the PCR amplicons. The raw chromatogram of the sequences was analyzed using MEGA11 version 11.0.13 [18] and assembled into a consensus sequence. The sequences were compared with other *O. tsutsugamushi* strains, *Candidatus* O. chuto, and *Candidatus* O. chiloeensis sequences that were retrieved from GenBank and aligned using MEGA11 with the MUSCLE (MUltiple Sequence Comparison by Log-Expectation) algorithm. Additionally, to infer the phylogenetic relationships among the sequences, we used a tool to find the best-fit model with the lowest Bayesian information criterion for nucleotide substitutions. Phylogenetic trees were constructed using the maximum-likelihood method with the General Time Reversible and Tamura 3-parameter models for the 56-kDa *tsa* and 47-kDa *htrA* gene partial sequences, respectively. Bootstrap analysis was replicated 1000 times to support the tree topology. Furthermore, sequence identity matrices were constructed using BioEdit version 7.7.1, using the acquired consensus sequences and reference sequences obtained from GenBank.

## 3. Results

### 3.1. Chigger Identification and Orientia Nucleotide Screening

A total of 1249 chiggers belonging to four genera and 14 species were identified. The predominant species was *Leptotrombidium pallidum* (59.6%), followed by *L. scutellare* (14.3%), *Neotrombicula kwangneungensis* (6.2%), *L. orientale* (4.5%), *Eushoengastia koreaensis* (4.4%), *L. palpale* (4.2%), *N. tamiyai* (3.1%), *N. japonica* (1.8%), and others (2%). *N. gardellai*, *N. nagayoi*, *L. gemiticulum*, *L. zetum*, *Cheladonta ikaoensis*, and *L. talmiensis* were rare species, accounting for less than 1% of the total.

The identified individuals were pooled into 266 pools, and seven pools (2.63%) were positive in the genus-specific quantitative real-time PCR assay. Among the positive samples, six pools were produced from *L. pallidum* and one pool was produced from *L. scutellare*, and all host rodents were *Apodemus agrarius*.

### 3.2. DNA Sequences of Orientia tsutsugamushi and Phylogenetic Analysis

The 56-kDa *tsa* and 47-kDa *htrA* gene fragments were obtained, respectively, from two and four pools in the seven *Orientia*-positive samples by qPCR targeting 16s RNA. In addition, these fragments were obtained from 19 and 20 pools, respectively, among the 21 pools of *O. tsutsugamushi*-positive samples in a previous survey conducted from 2022 to 2023. The lengths of the clean reads were 775–822 nucleotides for 56-kDa *tsa* and 823–853 nucleotides for 47-kDa *htrA*.

The representative sequence data of the 56-kDa *tsa* and 47-kDa *htrA* genes obtained in this study have been selected and deposited in the GenBank database. The accession numbers of 56-kDa *tsa* are PQ619408 to PQ619411, PQ627874, and PQ627876, and the accession numbers of 47-kDa *htrA* are PQ619412 to PQ619415, PQ627875, and PQ627877.

**Figure 2:**
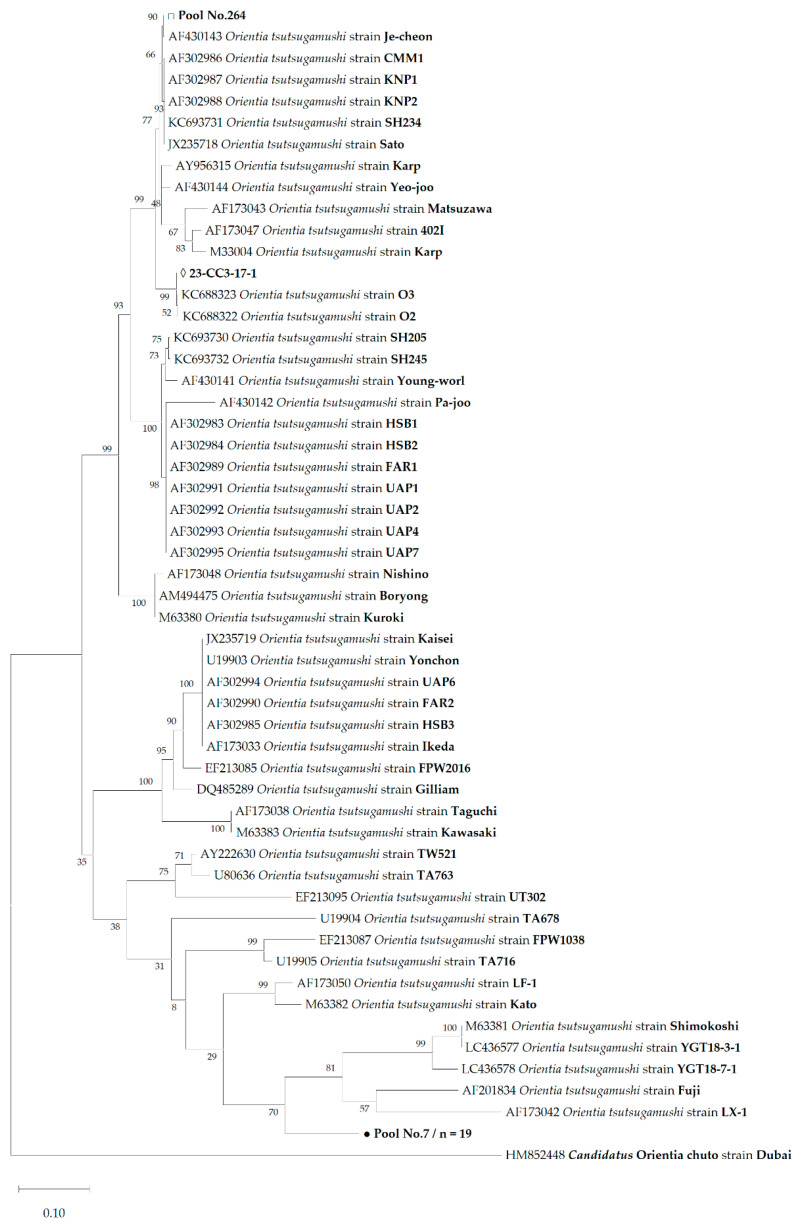
Phylogenetic analysis based on the 783 positions of the 56-kDa type-specific antigen using the Maximum Likelihood (ML) method based on the General Time Reversal model. The numbers on the branches indicate bootstrap percentages based on 1000 replications. The sequences identified as Boseong (GenBank accession numbers: PQ619408 to PQ619411), O3-related (GenBank accession number: PQ627876), and Je-cheon (GenBank accession number: PQ627874) strains in this study are indicated by black circles (●), white diamonds (◊), and white squares (□), respectively. The number (n) of sequences with an identical sequence is shown if the sequence was detected in more than one case.

**Figure 3:**
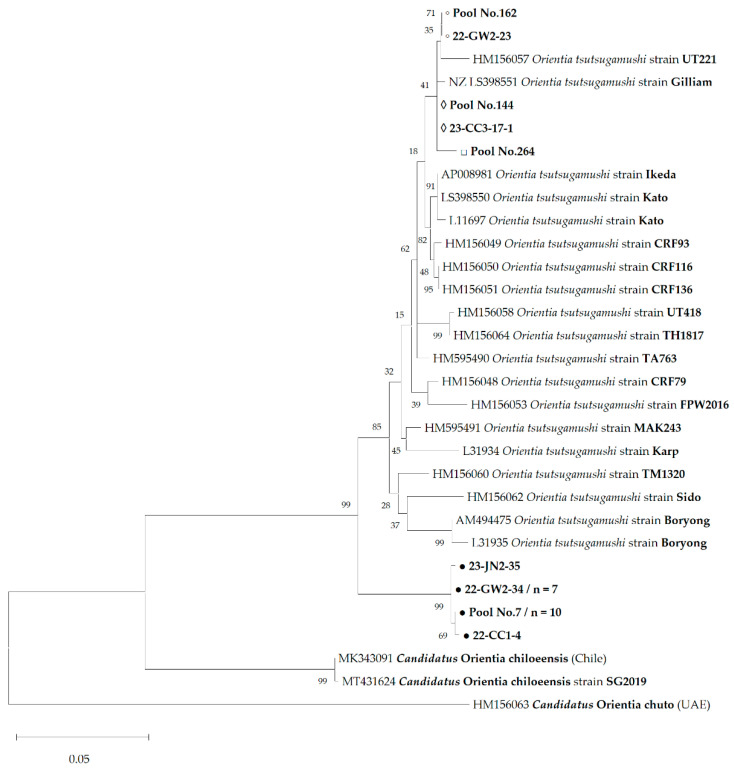
Phylogenetic analysis based on the 739 positions of 47-kDa high temperature requirement A using the Maximum Likelihood (ML) method based on the Tamura–3 parameter model. The numbers on the branches indicate bootstrap percentages based on 1000 replications. The sequences identified as Boseong (GenBank accession numbers: PQ619412 to PQ619415), O3-related (GenBank accession number: PQ627877), and Je-cheon (GenBank accession number: PQ627875) strains in this study are indicated by black circles (●), white diamonds (◊), and white squares (□), respectively. The unidentified sequences are indicated by a small white bullet (◦). The number (n) of sequences with an identical sequence is shown if the sequence was detected in more than one case.

In the phylogenetic analysis of 21 pools based on the 56-kDa *tsa* gene, 19 sequences formed a unique clade that clustered with highly divergent strains, such as strain Shimokoshi, supported by a 70% confidence value within the same species (Figure 2). Similarly, phylogenetic analysis based on the 47-kDa *htrA* gene amplicons of the same samples grouped into a single cluster with a 99% bootstrap value that was distinct from other reference strains (Figure 3). In addition, we confirmed the sequence of strain Je-cheon, which supported an 89% confidence value for the 56-kDa *tsa* tree. Similarly, we identified the sequence that formed a cluster with the O3 and O2 strains, which supported a 99% confidence value (Figure 2). In contrast, two sequences that could not be obtained from the 56-kDa *tsa* gene clustered with the UT221 strain in 47-kDa *htrA*.

The sequence identity of the amplified fragment was evaluated using matrices with other strains of *O. tsutsugamushi*, *Candidatus* O. chuto, and *Candidatus* O. chiloeensis. The 56-kDa *tsa* gene sequence of the new *O. tsutsugamushi* strain showed 63.2–77.8% similarity to other strains of the same species, and 55.6% similarity to *Candidatus* O. chuto (Supplementary Table S1). *Candidatus* O. chiloeensis could not be compared because of the lack of a known 56-kDa *tsa* gene sequence. Additionally, the 47-kDa *htrA* gene sequence of the new *O. tsutsugamushi* strain showed 99.5–100.0% similarity, within a maximum divergence of two nucleotides. In addition, it showed 93.2–94.7% similarity with the sequences of other known strains of *O. tsutsugamushi*, 82.6–83.5% with *Candidatus* O. chuto, and 87.6–88.3% with *Candidatus* O. chiloeensis (Supplementary Table S2).

## 4. Discussion

Antigenic variation in *Orientia* strains shows great inter-strain variability in virulence between humans and rodents, ranging from unapparent disease to fatality when untreated [3]. Therefore, obtaining information on the antigenicity and genetic variability of *Orientia* strains that are prevalent in endemic areas is an important step in vaccine development and diagnostic testing. Since 2010, new species have been reported in areas outside the Tsutsugamushi triangle [8,12], and most recently, the nucleotide sequence of *O. tsutsugamushi* has been detected in free-living chiggers in North America [13]. Under these circumstances, there is a need to re-examine the possibility of the existence of new or unrecorded *Orientia* in the ROK, where scrub typhus is prevalent, with more than 5000 cases reported annually from 2019 to 2023 [19].

Although no new species of *Orientia* were identified in this study, we identified novel strains of *O. tsutsugamushi*, namely, Boseong, Je-cheon, O2 and O3, based on genetic differences and a phylogenetic analysis of the 56-kDa *tsa* and 47-kDa *htrA* genes. Interestingly, the sequences of 396 nucleotides in the 56-kDa *tsa* gene of the Kato-related strain and previously [14] registered to GenBank (accession No. MZ146364 and MZ146358) were identical to those of the strain in this study, except for a difference in two nucleotides. Because our previous study had limitations due to short nucleotide sequences, this study complements the previous study and designates the novel strain Boseong, from where it originated. However, new strains have been confirmed not only in the Boseong area, which is in the southern region, but also in the northern and central regions of the ROK (Figure 1).

This means that the Boseong strain had already spread throughout the ROK, even though it was first identified in this study. Furthermore, we found sequences clustered with strain Je-cheon and a cluster of O3 and O2 on the 56-kDa *tsa* gene tree (Figure 2). These sequences showed >99% similarity to each reference strain. Nevertheless, further studies, such as genetic and antigenic characterizations, are required to determine whether these strains are identical. Meanwhile, Pool No. 162 and 22-GW2-23 could only amplify the sequence of the 47-kDa *htrA* gene through PCR and formed a cluster with the UT221 strain in the phylogenetic tree (Figure 3). Since 56-kDa *tsa* is the most widely used genetic marker for identifying strain heterogeneity [3], these two samples needed to be amplified for additional sequences with other primers or methods.

In the ROK, a nationwide survey on the prevalence of *O. tsutsugamushi* in wild rodents and trombiculid mites conducted from 2011 to 2013 found that strain Boryong was dominant at 85.6%, followed by Young-worl (3.8%), Je-cheon (3.4%), Yonchon (1.9%), TA763 (1.5%), pa-joo (1.1%), Kato (0.8%), KM02 (0.8%), Yeo-joo (0.4%), Ikeda (0.4%), and O107 (0.4%) [20]. The major strain of *O. tsutsugamushi* detected in patients with scrub typhus at the three regional hospitals was Boryong, accounting for 85.3% in 2014 and 90.4% in 2015 [21]. Moreover, Park et al. [22] reported that the Boryong strain was predominant in the southern region of the ROK, with *O. tsutsugamushi*-positive samples from chiggers and patients with scrub typhus collected between 2014 and 2016 showing a prevalence of 95.8% and 82.4%, respectively. In contrast, our previous study conducted on trombiculid mites harvested from wild rodents in 2020 showed a different pattern, with Kato-related strains, including the novel strain identified in this study (52.2%), being the most prevalent, followed by Karp-related strains (17.4%), Boryong (13.0%), JG-related (8.7%), Shimokoshi (4.3%), and Kawasaki (4.3%) [14]. In this study, we identified new and previously unreported strains in the ROK. These changes need to be examined in relation to the occurrence of scrub typhus. If the strains detected in chiggers and patients differ, further research on the virulence of each strain is necessary.

Previously, determining the infection rate of *O. tsutsugamushi* by trombiculid mite species was difficult, because slide-mounted specimens for identification were not available for use in other experiments. Therefore, research on the infection rate of *O. tsutsugamushi* by trombiculid mite species in wild rodents in the ROK is limited [22,23,24,25,26,27,28]. To solve this problem, we adopted autofluorescence microscopy for the morphological identification of trombiculid mites, as proposed by Kumlert et al. [15]. A clear advantage of this method is that morphological characteristics are easier to distinguish than when using a brightfield microscope, and specimens can be used for other purposes, such as molecular analysis, without having to mount slides for the purpose of identification. The 56-kDa *tsa* and 47-kDa *htrA* gene sequences of the positive samples were obtained from *L. pallidum*. Therefore, we believe that these methods will be useful for studying the prevalence and the main vectors of *Orientia* infection in various chigger species, including the newly identified Boseong strain.

This study has several limitations. Initially, only the phylogenetic analysis results were available for the 56-kDa *tsa* gene sequences clustered with the Je-cheon, O3, and O2 strains. In a study conducted in Japan [29], Saitama-type strains were distinguished into seven strains by antibody reactivity, even though they had 99.8% homologous 56-kDa *tsa* gene sequences. Therefore, additional research, such as serological tests or obtaining additional sequences of other genes with reference sequences, is necessary to supplement the results of this study. Secondly, 16S rRNA gene-based data were not included in this study. The 16S rRNA gene is widely used to investigate the taxonomy and evolution of prokaryotes [30]. We used primers to amplify the 16S rRNA gene from the novel strain [12,31], but we were unable to obtain an amplicon of the target. To overcome this problem, it is necessary to construct a 16S metagenomic sequencing library [32] along with other studies [11,13], or perform whole-genome analysis based on targeted enrichment sequencing after isolation [33]. Nonetheless, the 56-kDa *tsa* and 47-kDa *htrA* genes of the new strain in this study showed higher diversity than the other strains of *O. tsutsugamushi*. The final limitation of this study is that the results were obtained from trombiculid mites feeding on wild rodents. Because we used engorged chiggers harvested from wild rodents, the partial gene sequence of *O. tsutsugamushi* may have originated from the host itself. Further studies are needed to determine whether trombiculid mites can actually serve as a vector.

In conclusion, we identified a novel strain and a new record of the estimated O3 strain of *O. tsutsugamushi* based on the phylogenetic analysis of 56-kDa *tsa* and 47-kDa *htrA* gene sequences in the ROK. These findings provide additional confirmation of the pathogen that may cause scrub typhus in the ROK, and emphasize the need for continued surveillance and further research. In particular, the virulence, patient occurrence status, and epidemic potential of the Boseong strain observed in this study should be verified.

## Figures and Tables

**Table 1:** Collection information of *Orientia*-positive pools that obtained *tsa* and *htrA* gene sequences.

Sample No.	Site	Rodent Species	Chigger Species	No. of Specimens/Pool
7	Cheorwon	*Apodemus agrarius*	*Leptotrimbidium pallidum*	5
144	Paju			10
162	Paju			10
264	Yesan			5
22-GW1-5	Cheorwon	*Apodemus agrarius*	Unidentified	Minimum 1 to Maximum 30
22-GW1-18	Cheorwon			
22-GW2-23	Gangneung			
22-GW2-34	Gangneung			
22-SD2-12	Paju			
22-SD2-15	Paju			
22-SD2-22	Paju			
22-JN2-2	Boseong			
22-JN2-22	Boseong			
22-JN2-25	Boseong			
22-CC1-4	Cheongju			
23-SD2-16	Paju			
23-SD2-18	Paju			
23-JN2-6	Boseong			
23-JN2-8	Boseong			
23-JN2-23	Boseong			
23-JN2-35	Boseong			
23-JB-22	Jinan			
23-JB-26	Jinan			
23-CC3-17-1	Yesan			
23-CC3-17-2	Yesan			

**Table 2:** Primers and amplification conditions of semi-nested and nested polymerase chain reaction.

Target	Primer ID	Sequence (5′-3′)	Annealing Temperature	Reference
56-kDa *tsa*	Otr56_498F ^1^ r56_1459R ^1,2^ Otr56_585F ^1^	AATTAGTTTAGAATGGTTACCAC TCTGTATCTGTTCGACAGATGCACTATTA GAATGTCTGCGTTGTCGTTGC	54 °C	[12]
47-kDa *htrA*	Otr47_145F ^1^ Otr47_1780R ^1^ Otr47_263F ^2^ Otr47_1133R ^2^	ACAGGCCAAGATATTGGAAG AATCGCCTTTAAACTAGATTTACTTATTA GTGCTAAGAAARGATGATACTTC ACATTTAACATACCACGACGAAT	51 °C	[11]

^1^ 1st PCR; ^2^ 2nd PCR

## Data Availability

Data supporting the conclusions of this article are included within the article. The newly generated sequences were submitted to the GenBank database under the accession numbers PQ619408–PQ619415, and PQ627874–PQ627877. The datasets used and/or analyzed during the present study are available from the corresponding author upon reasonable request.

## Supplementary Materials

The following supporting information can be downloaded at: https://www.mdpi.com/article/10.3390/pathogens14010029/s1, Table S1: The identity matrix of 56-kDa *tsa* gene; Table S2: The identity matrix of 47-kDa *htrA* gene.
