# Supplementary material for: A Novel Strain of Orientia tsutsugamushi Detected from Chiggers (Acari: Trombiculidae) on Wild Rodents
Source: Pathogens. 2025 Jan 3;14(1):29. doi: 10.3390/pathogens14010029 (PMC11768423; doi:10.3390/pathogens14010029)
Supplement: Supplementary file 1 [file pathogens-14-00029-s001.zip › pathogens-3371258-supplementary.pdf]

Table S1. The identity matrix of 56-kDa *tsa* gene.

**Sequences**	**1**	**2**	**3**	**4**	**5**	**6**	**7**	**8**	**9**	**10**	**11**	**12**
**1. Pool No.7**	-											
**2. 23-CC3-17-1**	68.3	-										
**3. Pool No.264**	68.5	96.2	-									
**4.** AY956315 *Orientia tsutsugamushi* strain **CDC Karp**	69.6	95.6	97.6	-								
**5.** M33004 *Orientia tsutsugamushi* strain **Karp**	69.3	91.1	92.8	95.2	-							
**6.** DQ485289 *Orientia tsutsugamushi* strain **Gilliam**	71.5	78.3	78.8	78.4	77.0	-						
**7.** M63382 *Orientia tsutsugamushi* strain **Kato**	76.9	72.8	72.8	72.5	72.0	73.7	-					
**8.** U19904 *Orientia tsutsugamushi* strain **TA678**	70.0	71.8	73.1	72.7	71.8	69.1	74.6	-				
**9.** U80636 *Orientia tsutsugamushi* strain **TA763**	73.8	77.7	77.7	77.4	75.3	79.1	75.0	70.5	-			
**10.** U19905 *Orientia tsutsugamushi* strain **TA716**	76.9	71.8	72.0	71.7	70.5	72.5	79.2	74.5	81.3	-		
**11.** M63380 *Orientia tsutsugamushi* strain **Kuroki**	69.8	88.7	89.6	89.5	85.5	82.4	72.8	73.9	80.2	74.0	-	
**12.** M63381 *Orientia tsutsugamushi* strain **Shimokoshi**	74.4	68.9	68.5	69.1	68.5	68.4	71.0	69.7	68.1	69.7	69.9	-
**13.** AF173033 *Orientia tsutsugamushi* strain **Ikeda**	69.8	77.7	78.6	77.7	75.9	93.9	74.3	70.2	78.5	72.7	78.6	68.2
**14.** M63383 *Orientia tsutsugamushi* strain **Kawasaki**	73.3	74.2	74.6	74.8	73.2	86.6	74.5	70.1	75.7	71.6	73.8	70.2
**15.** AM494475 *Orientia tsutsugamushi* strain **Boryong**	69.8	88.7	89.6	89.5	85.5	82.4	72.8	73.9	80.2	74.0	100	69.9
**16.** U19903 *Orientia tsutsugamushi* strain **Yonchon**	69.8	77.7	78.6	77.7	75.9	93.9	74.3	70.2	78.5	72.7	78.6	68.2
**17.** AF430144 *Orientia tsutsugamushi* strain **yeo-joo**	68.3	95.1	97.8	97.3	92.6	77.5	72.5	73.1	76.9	71.1	88.9	68.2
**18.** AF430143 *Orientia tsutsugamushi* strain **je-cheon**	68.3	96.0	99.8	97.5	92.7	78.7	72.7	73.0	77.6	71.8	89.5	68.3
**19.** AF430142 *Orientia tsutsugamushi* strain **pa-joo**	63.2	85.1	85.2	84.7	80.4	72.1	67.9	67.8	71.7	65.9	83.4	64.5
**20.** AF430141 *Orientia tsutsugamushi* strain **young-worl**	66.3	86.4	86.9	86.5	82.7	73.9	70.1	71.5	73.6	67.9	85.0	67.2
**21.** AF173038 *Orientia tsutsugamushi* strain **Taguchi**	73.3	74.3	74.8	74.9	73.3	86.8	74.5	70.3	75.7	71.6	73.9	70.3
**22.** AF173042 *Orientia tsutsugamushi* strain **LX-1**	72.2	69.9	69.8	70.3	69.0	66.6	70.0	71.5	70.8	71.6	71.3	75.8
**23.** AF173043 *Orientia tsutsugamushi* strain **Matsuzawa**	67.6	90.9	93.4	93.0	93.7	76.7	72.2	72.1	74.6	69.8	85.7	68.2
**24.** AF173047 *Orientia tsutsugamushi* strain **402I**	68.2	91.7	94.9	92.8	96.9	78.0	72.8	72.5	76.3	71.4	85.9	68.2
**25.** AF173048 *Orientia tsutsugamushi* strain **Nishino**	69.1	87.9	88.6	88.3	84.4	81.7	73.0	73.3	80.0	74.2	98.8	69.5
**26.** AF173050 *Orientia tsutsugamushi* strain **LF-1**	77.8	73.0	72.1	70.9	70.4	74.3	93.4	75.1	74.4	78.7	71.7	70.4
**27.** AF201834 *Orientia tsutsugamushi* strain **Fuji**	76.1	67.6	67.3	68.2	67.3	66.8	69.1	70.1	70.1	74.3	69.6	74.7
**28.** AF302983 *Orientia tsutsugamushi* strain **HSB1**	68.1	91.1	91.1	90.6	86.3	77.5	73.1	73.1	77.0	71.1	89.0	69.2
**29.** AF302984 *Orientia tsutsugamushi* strain **HSB2**	68.1	91.1	91.1	90.6	86.3	77.5	73.1	73.1	77.0	71.1	89.0	69.2
**30.** AF302985 *Orientia tsutsugamushi* strain **HSB3**	69.8	77.7	78.6	77.7	75.9	93.9	74.3	70.2	78.5	72.7	78.6	68.2

**31.** AF302986 *Orientia tsutsugamushi* strain **CMM1**	68.5	96.0	99.5	97.5	92.7	79.0	73.3	73.4	78.2	72.4	89.8	68.6
**32.** AF302987 *Orientia tsutsugamushi* strain **KNP1**	68.5	96.0	99.5	97.5	92.7	79.0	73.3	73.4	78.2	72.4	89.8	68.6
**33.** AF302988 *Orientia tsutsugamushi* strain **KNP2**	68.5	96.0	99.5	97.5	92.7	79.0	73.3	73.4	78.2	72.4	89.8	68.6
**34.** AF302989 *Orientia tsutsugamushi* strain **FAR1**	68.1	91.1	91.1	90.6	86.3	77.5	73.1	73.1	77.0	71.1	89.0	69.2
**35.** AF302990 *Orientia tsutsugamushi* strain **FAR2**	69.8	77.7	78.6	77.7	75.9	93.9	74.3	70.2	78.5	72.7	78.6	68.2
**36.** AF302991 *Orientia tsutsugamushi* strain **UAP1**	68.1	91.1	91.1	90.6	86.3	77.5	73.1	73.1	77.0	71.1	89.0	69.2
**37.** AF302992 *Orientia tsutsugamushi* strain **UAP2**	68.1	91.1	91.1	90.6	86.3	77.5	73.1	73.1	77.0	71.1	89.0	69.2
**38.** AF302993 *Orientia tsutsugamushi* strain **UAP4**	68.1	91.1	91.1	90.6	86.3	77.5	73.1	73.1	77.0	71.1	89.0	69.2
**39.** AF302994 *Orientia tsutsugamushi* strain **UAP6**	69.8	77.7	78.6	77.7	75.9	93.9	74.3	70.2	78.5	72.7	78.6	68.2
**40.** AF302995 *Orientia tsutsugamushi* strain **UAP7**	68.1	91.1	91.1	90.6	86.3	77.5	73.1	73.1	77.0	71.1	89.0	69.2
**41.** AY222630 *Orientia tsutsugamushi* strain **TW521**	74.3	80.0	78.3	78.4	76.3	78.8	76.2	70.7	96.8	80.8	80.0	68.2
**42.** EF213085 *Orientia tsutsugamushi* strain **FPW2016**	66.3	75.0	74.6	73.2	71.4	88.9	70.6	65.7	73.6	67.2	74.1	64.8
**43.** EF213087 *Orientia tsutsugamushi* strain **FPW1038**	71.5	67.5	68.0	68.2	67.0	67.1	74.7	71.0	76.8	91.4	68.5	65.0
**44.** EF213095 *Orientia tsutsugamushi* strain **UT302**	72.3	73.7	72.0	71.3	69.6	70.9	73.1	69.6	81.2	77.8	72.5	67.3
**45.** JX235718 *Orientia tsutsugamushi* strain **Sato**	68.5	96.0	99.5	97.5	92.7	79.0	73.3	73.4	78.2	72.4	89.8	68.6
**46.** JX235719 *Orientia tsutsugamushi* strain **Kaisei**	69.8	77.7	78.6	77.7	75.9	93.9	74.3	70.2	78.5	72.7	78.6	68.2
**47.** KC688322 *Orientia tsutsugamushi* strain **O2**	68.3	99.7	96.2	95.3	90.8	78.1	72.8	71.8	77.7	71.8	88.7	68.8
**48.** KC688323 *Orientia tsutsugamushi* strain **O3**	68.5	99.8	96.0	95.4	91.0	78.1	73.0	72.0	77.9	72.0	88.9	68.9
**49.** KC693730 *Orientia tsutsugamushi* strain **SH205**	66.1	86.9	87.5	87.1	82.9	74.3	69.5	71.0	73.8	67.9	85.2	66.5
**50.** KC693731 *Orientia tsutsugamushi* strain **SH234**	68.5	96.0	99.5	97.5	92.7	79.0	73.3	73.4	78.2	72.4	89.8	68.6
**51.** KC693732 *Orientia tsutsugamushi* strain **SH245**	66.1	86.8	87.3	86.9	82.8	74.2	69.7	71.1	73.7	68.0	85.1	66.3
**52.** LC436577 *Orientia tsutsugamushi* strain **YGT18-3-1**	74.4	68.9	68.5	69.1	68.5	68.4	71.0	69.7	68.1	69.7	69.9	100
**53.** LC436578 *Orientia tsutsugamushi* strain **YGT18-7-1**	76.5	67.5	67.0	67.6	67.3	69.6	71.4	68.4	69.0	70.8	68.6	89.6
**54.** HM852448 ***Candidatus* Orientia chuto** strain **Dubai**	55.6	55.3	55.0	54.5	53.4	55.3	54.8	52.7	55.3	57.5	56.0	58.2

Table S1. (Continue)

Sequences	13	14	15	16	17	18	19	20	21	22	23	24
**13.** AF173033 *Orientia tsutsugamushi* strain **Ikeda**	-											
**14.** M63383 *Orientia tsutsugamushi* strain **Kawasaki**	86.8	-										
**15.** AM494475 *Orientia tsutsugamushi* strain **Boryong**	78.6	73.8	-									
**16.** U19903 *Orientia tsutsugamushi* strain **Yonchon**	100	86.8	78.6	-								
**17.** AF430144 *Orientia tsutsugamushi* strain **yeo-joo**	77.5	74.9	88.9	77.5	-							
**18.** AF430143 *Orientia tsutsugamushi* strain **je-cheon**	78.4	74.5	89.5	78.4	97.6	-						
**19.** AF430142 *Orientia tsutsugamushi* strain **pa-joo**	72.0	68.8	83.4	72.0	84.5	85.1	-					
**20.** AF430141 *Orientia tsutsugamushi* strain **young-worl**	73.6	70.8	85.0	73.6	86.4	86.8	87.2	-				
**21.** AF173038 *Orientia tsutsugamushi* strain **Taguchi**	86.6	99.8	73.9	86.6	75.1	74.6	68.9	71.0	-			
**22.** AF173042 *Orientia tsutsugamushi* strain **LX-1**	65.6	67.9	71.3	65.6	69.5	69.6	65.3	67.6	68.1	-		
**23.** AF173043 *Orientia tsutsugamushi* strain **Matsuzawa**	76.2	73.9	85.7	76.2	95.6	93.3	81.0	83.4	74.0	68.5	-	
**24.** AF173047 *Orientia tsutsugamushi* strain **402I**	77.4	73.6	85.9	77.4	93.0	94.7	81.1	83.4	73.7	68.8	94.3	-
**25.** AF173048 *Orientia tsutsugamushi* strain **Nishino**	78.0	73.2	98.8	78.0	87.7	88.5	82.8	84.1	73.3	71.3	84.7	84.9
**26.** AF173050 *Orientia tsutsugamushi* strain **LF-1**	73.7	74.8	71.7	73.7	71.2	72.0	66.8	69.3	74.8	69.4	70.8	72.0
**27.** AF201834 *Orientia tsutsugamushi* strain **Fuji**	66.9	69.1	69.6	66.9	67.5	67.2	63.5	66.1	68.9	77.4	66.5	67.0
**28.** AF302983 *Orientia tsutsugamushi* strain **HSB1**	77.2	73.9	89.0	77.2	90.5	90.9	93.5	93.3	74.0	70.6	87.0	87.1
**29.** AF302984 *Orientia tsutsugamushi* strain **HSB2**	77.2	73.9	89.0	77.2	90.5	90.9	93.5	93.3	74.0	70.6	87.0	87.1
**30.** AF302985 *Orientia tsutsugamushi* strain **HSB3**	100	86.8	78.6	100	77.5	78.4	72.0	73.6	86.6	65.6	76.2	77.4
**31.** AF302986 *Orientia tsutsugamushi* strain **CMM1**	78.7	74.8	89.8	78.7	97.6	99.4	85.2	87.1	74.9	69.9	93.3	95.3
**32.** AF302987 *Orientia tsutsugamushi* strain **KNP1**	78.7	74.8	89.8	78.7	97.6	99.4	85.2	87.1	74.9	69.9	93.3	95.3
**33.** AF302988 *Orientia tsutsugamushi* strain **KNP2**	78.7	74.8	89.8	78.7	97.6	99.4	85.2	87.1	74.9	69.9	93.3	95.3
**34.** AF302989 *Orientia tsutsugamushi* strain **FAR1**	77.2	73.9	89.0	77.2	90.5	90.9	93.5	93.3	74.0	70.6	87.0	87.1
**35.** AF302990 *Orientia tsutsugamushi* strain **FAR2**	100	86.8	78.6	100	77.5	78.4	72.0	73.6	86.6	65.6	76.2	77.4
**36.** AF302991 *Orientia tsutsugamushi* strain **UAP1**	77.2	73.9	89.0	77.2	90.5	90.9	93.5	93.3	74.0	70.6	87.0	87.1
**37.** AF302992 *Orientia tsutsugamushi* strain **UAP2**	77.2	73.9	89.0	77.2	90.5	90.9	93.5	93.3	74.0	70.6	87.0	87.1
**38.** AF302993 *Orientia tsutsugamushi* strain **UAP4**	77.2	73.9	89.0	77.2	90.5	90.9	93.5	93.3	74.0	70.6	87.0	87.1
**39.** AF302994 *Orientia tsutsugamushi* strain **UAP6**	100	86.8	78.6	100	77.5	78.4	72.0	73.6	86.6	65.6	76.2	77.4
**40.** AF302995 *Orientia tsutsugamushi* strain **UAP7**	77.2	73.9	89.0	77.2	90.5	90.9	93.5	93.3	74.0	70.6	87.0	87.1
**41.** AY222630 *Orientia tsutsugamushi* strain **TW521**	79.1	76.5	80.0	79.1	77.6	78.2	72.7	74.3	76.5	70.9	75.3	76.9
**42.** EF213085 *Orientia tsutsugamushi* strain **FPW2016**	89.8	82.1	74.1	89.8	73.6	74.5	69.5	71.0	81.9	62.1	72.3	73.6

**43.** EF213087 *Orientia tsutsugamushi* strain **FPW1038**	68.7	68.4	68.5	68.7	67.9	67.9	65.6	64.6	68.4	66.9	66.6	67.5
**44.** EF213095 *Orientia tsutsugamushi* strain **UT302**	72.8	73.6	72.5	72.8	71.5	71.9	65.3	68.0	73.6	71.9	69.6	70.9
**45.** JX235718 *Orientia tsutsugamushi* strain **Sato**	78.7	74.8	89.8	78.7	97.6	99.4	85.2	87.1	74.9	69.9	93.3	95.3
**46.** JX235719 *Orientia tsutsugamushi* strain **Kaisei**	100	86.8	78.6	100	77.5	78.4	72.0	73.6	86.6	65.6	76.2	77.4
**47.** KC688322 *Orientia tsutsugamushi* strain **O2**	77.5	73.9	88.7	77.5	94.9	96.0	85.4	86.6	74.0	69.9	90.6	91.7
**48.** KC688323 *Orientia tsutsugamushi* strain **O3**	77.5	74.0	88.9	77.5	95.0	95.9	85.2	86.5	74.2	70.0	90.8	91.5
**49.** KC693730 *Orientia tsutsugamushi* strain **SH205**	74.0	71.1	85.2	74.0	86.9	87.3	88.3	97.7	71.2	67.6	83.6	83.8
**50.** KC693731 *Orientia tsutsugamushi* strain **SH234**	78.7	74.8	89.8	78.7	97.6	99.4	85.2	87.1	74.9	69.9	93.3	95.3
**51.** KC693732 *Orientia tsutsugamushi* strain **SH245**	73.9	71.0	85.1	73.9	86.8	87.2	88.4	97.8	71.1	67.7	83.4	83.6
**52.** LC436577 *Orientia tsutsugamushi* strain **YGT18-3-1**	68.2	70.2	69.9	68.2	68.2	68.3	64.5	67.2	70.3	75.8	68.2	68.2
**53.** LC436578 *Orientia tsutsugamushi* strain **YGT18-7-1**	69.0	70.1	68.6	69.0	67.0	66.9	62.7	65.8	70.2	73.7	67.0	67.2
**54.** HM852448 ***Candidatus*** **Orientia chuto** strain **Dubai**	54.7	54.8	56.0	54.7	55.0	54.8	51.9	53.9	55.0	55.3	54.1	54.3

Table S1. (Continue)

Sequences	25	26	27	28	29	30	31	32	33	34	35	36
**25.** AF173048 *Orientia tsutsugamushi* strain **Nishino**	-											
**26.** AF173050 *Orientia tsutsugamushi* strain **LF-1**	71.8	-										
**27.** AF201834 *Orientia tsutsugamushi* strain **Fuji**	69.2	68.4	-									
**28.** AF302983 *Orientia tsutsugamushi* strain **HSB1**	88.2	72.1	68.9	-								
**29.** AF302984 *Orientia tsutsugamushi* strain **HSB2**	88.2	72.1	68.9	100	-							
**30.** AF302985 *Orientia tsutsugamushi* strain **HSB3**	78.0	73.7	66.9	77.2	77.2	-						
**31.** AF302986 *Orientia tsutsugamushi* strain **CMM1**	88.6	72.4	67.8	91.2	91.2	78.7	-					
**32.** AF302987 *Orientia tsutsugamushi* strain **KNP1**	88.6	72.4	67.8	91.2	91.2	78.7	100	-				
**33.** AF302988 *Orientia tsutsugamushi* strain **KNP2**	88.6	72.4	67.8	91.2	91.2	78.7	100	100	-			
**34.** AF302989 *Orientia tsutsugamushi* strain **FAR1**	88.2	72.1	68.9	100	100	77.2	91.2	91.2	91.2	-		
**35.** AF302990 *Orientia tsutsugamushi* strain **FAR2**	78.0	73.7	66.9	77.2	77.2	100.0	78.7	78.7	78.7	77.2	-	
**36.** AF302991 *Orientia tsutsugamushi* strain **UAP1**	88.2	72.1	68.9	100	100	77.2	91.2	91.2	91.2	100	77.2	-
**37.** AF302992 *Orientia tsutsugamushi* strain **UAP2**	88.2	72.1	68.9	100	100	77.2	91.2	91.2	91.2	100	77.2	100
**38.** AF302993 *Orientia tsutsugamushi* strain **UAP4**	88.2	72.1	68.9	100	100	77.2	91.2	91.2	91.2	100	77.2	100
**39.** AF302994 *Orientia tsutsugamushi* strain **UAP6**	78.0	73.7	66.9	77.2	77.2	100	78.7	78.7	78.7	77.2	100	77.2
**40.** AF302995 *Orientia tsutsugamushi* strain **UAP7**	88.2	72.1	68.9	100	100	77.2	91.2	91.2	91.2	100	77.2	100
**41.** AY222630 *Orientia tsutsugamushi* strain **TW521**	79.4	75.9	70.1	78.0	78.0	79.1	78.7	78.7	78.7	78.0	79.1	78.0
**42.** EF213085 *Orientia tsutsugamushi* strain **FPW2016**	73.4	71.8	62.9	74.6	74.6	89.8	75.0	75.0	75.0	74.6	89.8	74.6
**43.** EF213087 *Orientia tsutsugamushi* strain **FPW1038**	68.6	73.8	70.4	67.6	67.6	68.7	68.5	68.5	68.5	67.6	68.7	67.6
**44.** EF213095 *Orientia tsutsugamushi* strain **UT302**	72.2	72.2	68.9	71.0	71.0	72.8	72.5	72.5	72.5	71.0	72.8	71.0
**45.** JX235718 *Orientia tsutsugamushi* strain **Sato**	88.6	72.4	67.8	91.2	91.2	78.7	100	100	100	91.2	78.7	91.2
**46.** JX235719 *Orientia tsutsugamushi* strain **Kaisei**	78.0	73.7	66.9	77.2	77.2	100	78.7	78.7	78.7	77.2	100	77.2
**47.** KC688322 *Orientia tsutsugamushi* strain **O2**	87.9	73.0	67.6	91.4	91.4	77.5	96.0	96.0	96.0	91.4	77.5	91.4
**48.** KC688323 *Orientia tsutsugamushi* strain **O3**	88.0	73.1	67.8	91.2	91.2	77.5	95.9	95.9	95.9	91.2	77.5	91.2
**49.** KC693730 *Orientia tsutsugamushi* strain **SH205**	84.7	68.8	66.3	94.5	94.5	74.0	87.8	87.8	87.8	94.5	74.0	94.5
**50.** KC693731 *Orientia tsutsugamushi* strain **SH234**	88.6	72.4	67.8	91.2	91.2	78.7	100	100	100	91.2	78.7	91.2
**51.** KC693732 *Orientia tsutsugamushi* strain **SH245**	84.5	68.7	66.5	94.6	94.6	73.9	87.6	87.6	87.6	94.6	73.9	94.6
**52.** LC436577 *Orientia tsutsugamushi* strain **YGT18-3-1**	69.5	70.4	74.7	69.2	69.2	68.2	68.6	68.6	68.6	69.2	68.2	69.2
**53.** LC436578 *Orientia tsutsugamushi* strain **YGT18-7-1**	68.1	71.2	74.7	67.6	67.6	69.0	67.3	67.3	67.3	67.6	69.0	67.6
**54.** HM852448 ***Candidatus* Orientia chuto** strain **Dubai**	55.6	54.3	54.9	55.6	55.6	54.7	55.1	55.1	55.1	55.6	54.7	55.6

Table S1. (Continue)

Sequences	37	38	39	40	41	42	43	44	45	46	47	48
**37.** AF302992 *Orientia tsutsugamushi* strain **UAP2**	-											
**38.** AF302993 *Orientia tsutsugamushi* strain **UAP4**	100	-										
**39.** AF302994 *Orientia tsutsugamushi* strain **UAP6**	77.2	77.2	-									
**40.** AF302995 *Orientia tsutsugamushi* strain **UAP7**	100	100	77.2	-								
**41.** AY222630 *Orientia tsutsugamushi* strain **TW521**	78.0	78.0	79.1	78.0	-							
**42.** EF213085 *Orientia tsutsugamushi* strain **FPW2016**	74.6	74.6	89.8	74.6	75.6	-						
**43.** EF213087 *Orientia tsutsugamushi* strain **FPW1038**	67.6	67.6	68.7	67.6	76.8	63.4	-					
**44.** EF213095 *Orientia tsutsugamushi* strain **UT302**	71.0	71.0	72.8	71.0	83.1	69.0	73.5	-				
**45.** JX235718 *Orientia tsutsugamushi* strain **Sato**	91.2	91.2	78.7	91.2	78.7	75.0	68.5	72.5	-			
**46.** JX235719 *Orientia tsutsugamushi* strain **Kaisei**	77.2	77.2	100	77.2	79.1	89.8	68.7	72.8	78.7	-		
**47.** KC688322 *Orientia tsutsugamushi* strain **O2**	91.4	91.4	77.5	91.4	80.0	74.9	67.5	73.7	96.0	77.5	-	
**48.** KC688323 *Orientia tsutsugamushi* strain **O3**	91.2	91.2	77.5	91.2	80.2	74.9	67.6	73.9	95.9	77.5	99.8	-
**49.** KC693730 *Orientia tsutsugamushi* strain **SH205**	94.5	94.5	74.0	94.5	74.5	71.4	64.6	67.9	87.8	74.0	87.2	87.1
**50.** KC693731 *Orientia tsutsugamushi* strain **SH234**	91.2	91.2	78.7	91.2	78.7	75.0	68.5	72.5	100	78.7	96.0	95.9
**51.** KC693732 *Orientia tsutsugamushi* strain **SH245**	94.6	94.6	73.9	94.6	74.4	71.3	64.7	68.0	87.6	73.9	87.1	86.9
**52.** LC436577 *Orientia tsutsugamushi* strain **YGT18-3-1**	69.2	69.2	68.2	69.2	68.2	64.8	65.0	67.3	68.6	68.2	68.8	68.9
**53.** LC436578 *Orientia tsutsugamushi* strain **YGT18-7-1**	67.6	67.6	69.0	67.6	69.4	65.6	66.2	67.5	67.3	69.0	67.3	67.5
**54.** HM852448 ***Candidatus* Orientia chuto** strain **Dubai**	55.6	55.6	54.7	55.6	55.6	52.0	53.1	55.7	55.1	54.7	55.1	55.3

Table S1. (Continue)

Sequences	49	50	51	52	53	54
**49.** KC693730 *Orientia tsutsugamushi* strain **SH205**	-					
**50.** KC693731 *Orientia tsutsugamushi* strain **SH234**	87.8	-				
**51.** KC693732 *Orientia tsutsugamushi* strain **SH245**	99.5	87.6	-			
**52.** LC436577 *Orientia tsutsugamushi* strain **YGT18-3-1**	66.5	68.6	66.3	-		
**53.** LC436578 *Orientia tsutsugamushi* strain **YGT18-7-1**	65.1	67.3	65.2	89.6	-	
**54.** HM852448 ***Candidatus* Orientia chuto** strain **Dubai**	53.6	55.1	53.8	58.2	60.5	-

Table S2. The identity matrix of 47-kDa *htrA* gene.

**Sequences**	**1**	**2**	**3**	**4**	**5**	**6**	**7**	**8**	**9**	**10**	**11**	**12**
**1. Pool No.7**	-											
**2. Pool No.144**	93.9	-										
**3. Pool No.162**	94.0	99.8	-									
**4. Pool No.264**	94.0	99.3	99.1	-								
**5. 22-GW2-34**	99.8	94.0	94.1	94.1	-							
**6. 23-JN2-35**	99.7	93.9	94.0	94.0	99.8	-						
**7. 22-CC1-4**	99.8	93.7	93.9	93.9	99.7	99.5	-					
**8. 23-CC3-17-1**	93.9	100	99.8	99.3	94.0	93.9	93.7	-				
**9. 22-GW2-23**	94.0	99.8	100	99.1	94.1	94.0	93.9	99.8	-			
**10.** L31934 *Orientia tsutsugamushi* strain **Karp**	93.9	97.1	96.9	97.1	94.0	93.9	93.7	97.1	96.9	-		
**11.** LS398551 *Orientia tsutsugamushi* strain **Gilliam**	93.7	99.7	99.5	99.0	93.9	93.7	93.6	99.7	99.5	96.9	-	
**12.** LS398550 *Orientia tsutsugamushi* strain **Kato**	94.1	99.1	99.0	98.4	94.3	94.1	94.0	99.1	99.0	97.1	98.9	-
**13.** L11697 *Orientia tsutsugamushi* strain **Kato**	93.9	98.9	98.7	98.2	94.0	93.9	93.7	98.9	98.7	96.8	98.6	99.7
**14.** HM595490 *Orientia tsutsugamushi* strain **TA763**	94.4	98.9	98.7	99.0	94.5	94.4	94.3	98.9	98.7	97.5	98.6	98.9
**15.** HM156064 *Orientia tsutsugamushi* strain **TH1817**	93.9	98.4	98.3	98.3	94.0	93.9	93.7	98.4	98.3	97.1	98.6	98.2
**16.** AP008981 *Orientia tsutsugamushi* strain **Ikeda**	94.1	99.1	99.0	98.4	94.3	94.1	94.0	99.1	99.0	97.1	98.9	100
**17.** AM494475 *Orientia tsutsugamushi* strain **Boryong**	93.6	96.4	96.3	96.8	93.7	93.6	93.5	96.4	96.3	96.1	96.3	96.4
**18.** L31935 *Orientia tsutsugamushi* strain **Boryong**	93.3	96.3	96.1	96.5	93.5	93.3	93.2	96.3	96.1	95.9	96.1	96.1
**19.** HM156062 *Orientia tsutsugamushi* strain **Sido**	93.7	96.1	96.3	96.3	93.9	93.7	93.6	96.1	96.3	95.4	95.9	96.3
**20.** HM156048 *Orientia tsutsugamushi* strain **CRF79**	94.0	98.3	98.2	98.2	94.1	94.0	93.9	98.3	98.2	97.4	98.0	98.3
**21.** HM156049 *Orientia tsutsugamushi* strain **CRF93**	94.1	99.0	98.9	98.3	94.3	94.1	94.0	99.0	98.9	97.1	98.7	99.5
**22.** HM156050 *Orientia tsutsugamushi* strain **CRF116**	93.9	99.1	99.0	98.4	94.0	93.9	93.7	99.1	99.0	97.1	98.9	99.4
**23.** HM156051 *Orientia tsutsugamushi* strain **CRF136**	93.9	99.1	99.0	98.4	94.0	93.9	93.7	99.1	99.0	97.1	98.9	99.4
**24.** HM156053 *Orientia tsutsugamushi* strain **FPW2016**	94.0	97.2	97.1	97.4	94.1	94.0	93.9	97.2	97.1	97.1	96.9	97.5
**25.** HM156057 *Orientia tsutsugamushi* strain **UT221**	93.9	98.9	99.0	98.4	94.0	93.9	93.7	98.9	99.0	96.3	98.9	98.0
**26.** HM156058 *Orientia tsutsugamushi* strain **UT418**	93.9	98.3	98.2	98.2	94.0	93.9	93.7	98.3	98.2	97.1	98.4	98.0
**27.** HM156060 *Orientia tsutsugamushi* strain **TM1320**	94.0	97.6	97.5	96.9	94.1	94.0	93.9	97.6	97.5	96.7	97.4	98.2
**28.** HM595491 *Orientia tsutsugamushi* strain **MAK243**	94.7	98.0	97.9	98.2	94.8	94.7	94.5	98.0	97.9	97.8	97.8	98.0
**29.** MK343091 ***Candidatus*** **Orientia chiloeensis** (Chile)	87.9	88.0	88.1	88.0	88.0	87.9	87.8	88.0	88.1	87.3	87.9	87.7
**30.** MT431624 ***Candidatus*** **Orientia chiloeensis** strain **SG2019** (Chile)	87.8	88.1	88.3	88.0	87.9	87.8	87.6	88.1	88.3	87.3	88.0	87.9

**31.** HM156063 ***Candidatus*** **Orientia chuto** (UAE)	82.8	83.4	83.5	83.5	82.9	82.8	82.6	83.4	83.5	83.0	83.5	83.1

Table S2. (Continue)

Sequences	13	14	15	16	17	18	19	20	21	22	23	24
**13.** L11697 *Orientia tsutsugamushi* strain **Kato**	-											
**14.** HM595490 *Orientia tsutsugamushi* strain **TA763**	98.6	-										
**15.** HM156064 *Orientia tsutsugamushi* strain **TH1817**	97.9	98.4	-									
**16.** AP008981 *Orientia tsutsugamushi* strain **Ikeda**	99.7	98.9	98.2	-								
**17.** AM494475 *Orientia tsutsugamushi* strain **Boryong**	96.1	96.9	96.4	96.4	-							
**18.** L31935 *Orientia tsutsugamushi* strain **Boryong**	95.9	96.4	96.1	96.1	99.4	-						
**19.** HM156062 *Orientia tsutsugamushi* strain **Sido**	96.0	96.4	95.4	96.3	96.7	96.1	-					
**20.** HM156048 *Orientia tsutsugamushi* strain **CRF79**	98.0	98.6	97.9	98.3	96.4	96.1	96.0	-				
**21.** HM156049 *Orientia tsutsugamushi* strain **CRF93**	99.3	98.7	98.0	99.5	96.4	96.1	96.3	98.3	-			
**22.** HM156050 *Orientia tsutsugamushi* strain **CRF116**	99.1	98.9	98.2	99.4	96.4	96.1	96.0	98.3	99.5	-		
**23.** HM156051 *Orientia tsutsugamushi* strain **CRF136**	99.1	98.9	98.2	99.4	96.4	96.1	96.0	98.3	99.5	100	-	
**24.** HM156053 *Orientia tsutsugamushi* strain **FPW2016**	97.2	97.8	96.8	97.5	96.9	96.4	96.8	98.3	97.8	97.5	97.5	-
**25.** HM156057 *Orientia tsutsugamushi* strain **UT221**	97.8	98.0	98.0	98.0	96.5	96.4	96.3	97.5	97.9	98.0	98.0	96.7
**26.** HM156058 *Orientia tsutsugamushi* strain **UT418**	97.8	98.3	99.8	98.0	96.4	96.1	95.4	97.9	98.2	98.0	98.0	96.8
**27.** HM156060 *Orientia tsutsugamushi* strain **TM1320**	97.9	97.4	96.9	98.2	97.4	97.1	96.9	97.2	97.9	97.9	97.9	97.5
**28.** HM595491 *Orientia tsutsugamushi* strain **MAK243**	97.8	98.3	97.6	98.0	97.2	96.9	96.7	98.0	98.0	98.0	98.0	97.5
**29.** MK343091 ***Candidatus* Orientia chiloeensis** (Chile)	87.5	87.9	87.6	87.7	87.6	87.6	88.3	87.5	87.7	87.5	87.5	87.5
**30.** MT431624 ***Candidatus* Orientia chiloeensis** strain **SG2019** (Chile)	87.6	87.9	87.6	87.9	87.6	87.6	88.3	87.5	87.9	87.6	87.6	87.5
**31.** HM156063 ***Candidatus* Orientia chuto** (UAE)	82.8	83.2	83.5	83.1	83.1	83.4	83.2	83.1	83.1	82.8	82.8	82.8

Table S2. (Continue)

Sequences	25	26	27	28	29	30	31
**25.** HM156057 *Orientia tsutsugamushi* strain **UT221**	-						
**26.** HM156058 *Orientia tsutsugamushi* strain **UT418**	97.9	-					
**27.** HM156060 *Orientia tsutsugamushi* strain **TM1320**	97.1	96.9	-				
**28.** HM595491 *Orientia tsutsugamushi* strain **MAK243**	97.6	97.6	97.6	-			
**29.** MK343091 ***Candidatus*** **Orientia chiloeensis** (Chile)	88.1	87.6	88.1	88.1	-		
**30.** MT431624 ***Candidatus*** **Orientia chiloeensis** strain **SG2019** (Chile)	88.1	87.6	88.3	88.1	99.7	-	
**31.** HM156063 ***Candidatus*** **Orientia chuto** (UAE)	83.5	83.5	83.0	83.6	83.9	83.9	-
